# Intercenter reliability and validity of the rhesus macaque GeneChip

**DOI:** 10.1186/1471-2164-8-61

**Published:** 2007-02-28

**Authors:** Fenghai Duan, Eliot R Spindel, Yu-Hua Li, Robert B Norgren

**Affiliations:** 1 Department of Preventive and Societal Medicine, University of Nebraska Medical Center, Omaha, NE, USA; 2 Division of Neuroscience, Oregon National Primate Research Center, Oregon Health & Science University, Beaverton, OR, USA; 3 Yerkes National Primate Research Center, Emory University, Atlanta, GA, USA; 4 Department of Genetics, Cell Biology and Anatomy, University of Nebraska Medical Center, Omaha, NE, USA

## Abstract

**Background:**

The non-human primate (NHP) research community has been intensely interested in obtaining whole-genome expression arrays for their work. Recently, novel approaches were used to generate the DNA sequence information for a rhesus GeneChip. To test the reliability of the rhesus GeneChip across different centers, RNA was isolated from five sources: cerebral cortex, pancreas, thymus, testis, and an immortalized fibroblast cell line. Aliquots of this RNA were sent to each of three centers: Yerkes National Primate Research Center, Oregon National Primate Research Center and the University of Nebraska Medical Center. Each center labeled the samples and hybridized them with two rhesus macaque GeneChips. In addition, rhesus samples were hybridzed with human GeneChips to compare with samples hybridized with the rhesus GeneChip.

**Results:**

The results indicate that center effects were minimal and the rhesus GeneChip appears highly reliable. To test the validity of the rhesus GeneChip, five of the most differentially expressed genes among tissues identified in the reliability experiments were chosen for analysis with Quantitative PCR. For all 5 genes, the qPCR and GeneChip results were in agreement with regard to differential expression between tissues. Significantly more probesets were called present when rhesus samples were hybridized with the rhesus GeneChip than when these same samples were hybridized with a human GeneChip.

**Conclusion:**

The rhesus GeneChip is both a reliable and a valid tool for examining gene expression and represents a significant improvement over the use of the human GeneChip for rhesus macaque gene expression studies.

## Background

The non-human primate (NHP) research community has been intensely interested in obtaining whole-genome expression arrays for their work. The recent production of a rhesus macaque GeneChip (Affymetrix, Santa Clara, CA) now satisfies this need [1].

Novel approaches were used to generate the DNA sequence information for the rhesus GeneChip. In 2005, when the rhesus macaque GeneChip was in the design stage, the percent of the total genes in the rhesus macaque genome covered by the ESTs was quite small. In addition, the rhesus macaque genome sequences were at an early stage of assembly and with limited redundancy. To overcome these limitations, we used a targeted PCR approach to acquire necessary sequences for the probes for over 5,000 genes [2]. All human last exons were identified and aligned with Probe Selection Region (PSR) sequences obtained from Affymetrix. Primers were designed that flanked the PSRs. These primers were used to amplify orthologous PSRs in rhesus macaques from rhesus genomic DNA. The PCR products were cloned, sequenced and deposited in GenBank. In an *in silico *version of our targeted PCR approach, sequences from an early draft of the Baylor Rhesus Genome assembly were aligned with human PSRs and information for probe design extracted. Using primarily these two sources of sequence information, a whole genome rhesus macaqe expression GeneChip was created by Affymetrix. The targeted acquisition of the 3' gene sequence information used for the production of the rhesus macaque GeneChip is unique among the Affymetrix GeneChips.

The rhesus macaque GeneChip uses 52,000 probesets to monitor the expression of over 47,000 transcripts, including transcripts with multiple polyadenylation sites. Reliability and reproducibility are major issues that must be addressed to successfully apply microarray technology to biomedical experiments [3,4]. They are particularly important when researchers want to compare and integrate microarray experiments from multiple laboratories [5-7]. Due to the time and expense associated with NHP experiments, it is critical that the results generated in one center be comparable with results at another.

Given the novel strategies underlying the design of the rhesus macaque GeneChip, we felt it was important to test its reliability and validity. To test the reliability of the rhesus GeneChip across different centers, RNA was isolated from five sources: cerebral cortex, pancreas, thymus, testis, and an immortalized fibroblast cell line. Two aliquots of RNA from each tissue type were sent to each of three centers: Yerkes National Primate Research Center, Oregon National Primate Research Center and the University of Nebraska Medical Center. Each center labeled the two aliquots individually and hybridized them to two separate rhesus macaque GeneChips. The results indicate that center effects were minimal and the rhesus GeneChip appears highly reliable. To test the validity of the rhesus GeneChip, five of the most differentially expressed genes among tissues identified in the reliability experiments were chosen for analysis with Quantitative PCR. The results indicated the rhesus GeneChip provides valid information.

Prior to the production of the rhesus GeneChip, many investigators used various human expression arrays with rhesus samples [8]. Although useful in obtaining some information, studies indicated that performing these cross-species hybridizations were resulting in the loss of considerable data [9,10]. We wanted to determine whether more probesets would be called present when rhesus samples were hybridized with the rhesus GeneChip in comparison to the human GeneChip. We found the rhesus GeneChip was superior to the human GeneChip for use with rhesus samples in all five tested samples.

## Results

### Quality assessment

**Figure 1:**
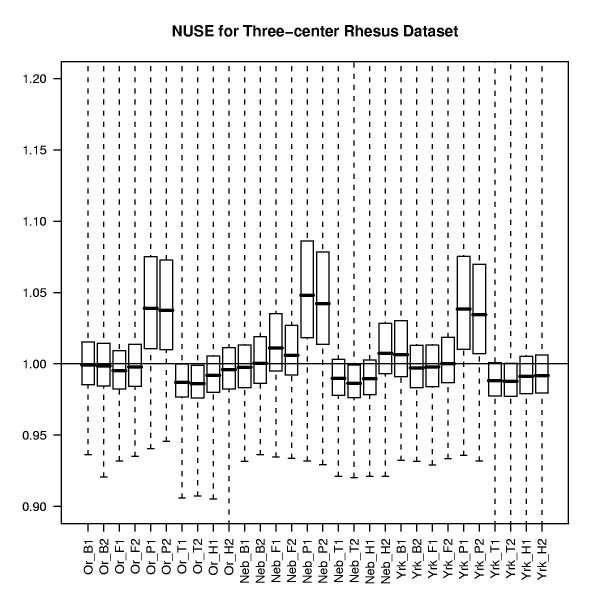
**NUSE boxplot. **After RMA preprocessing, the standard errors were standardized across arrays and then plotted to assess the data quality. x axis: the first letter "Or, Neb and Yrk" stands for three laboratories from the centers of Oregon, Nebraska and Yerkes, respectively. The second letter "B, F, P, T, H" represents the tissues of Brain (Cerebral Cortex), Fibroblast, Pancreas, Testis and Thymus, respectively. The third letter "1 and 2" are the two replicated measurements for each RNA extract.

Affymetrix GCOS quality control parameters – the background levels, the range of the present percentage, the scaling factor values, the 3'/5' ratios of housekeeping genes and the signal intensities of spiked-in hybridization controls were all within acceptable levels as described by Affymetrix for the data from the cerebral cortex, fibroblast, testis and thymus experiments. The scaling factor (Table 1) and the NUSE value (Figure 1) indicated that the data from the experiments involving pancreas samples was of lower quality than the experiments involving the other four tissues. The scaling factors of the pancreas arrays were larger than any of the other samples for each lab and some of the scaling factors for the pancreas results were three fold larger than the average, which is above the tolerance threshold suggested by Affymetrix. The NUSE medians and the interquartile ranges (IQR) of the pancreas arrays were larger than those of the other arrays, indicating that the data from the pancreas arrays should be treated with caution.

### Correlation analysis

**Figure 2:**
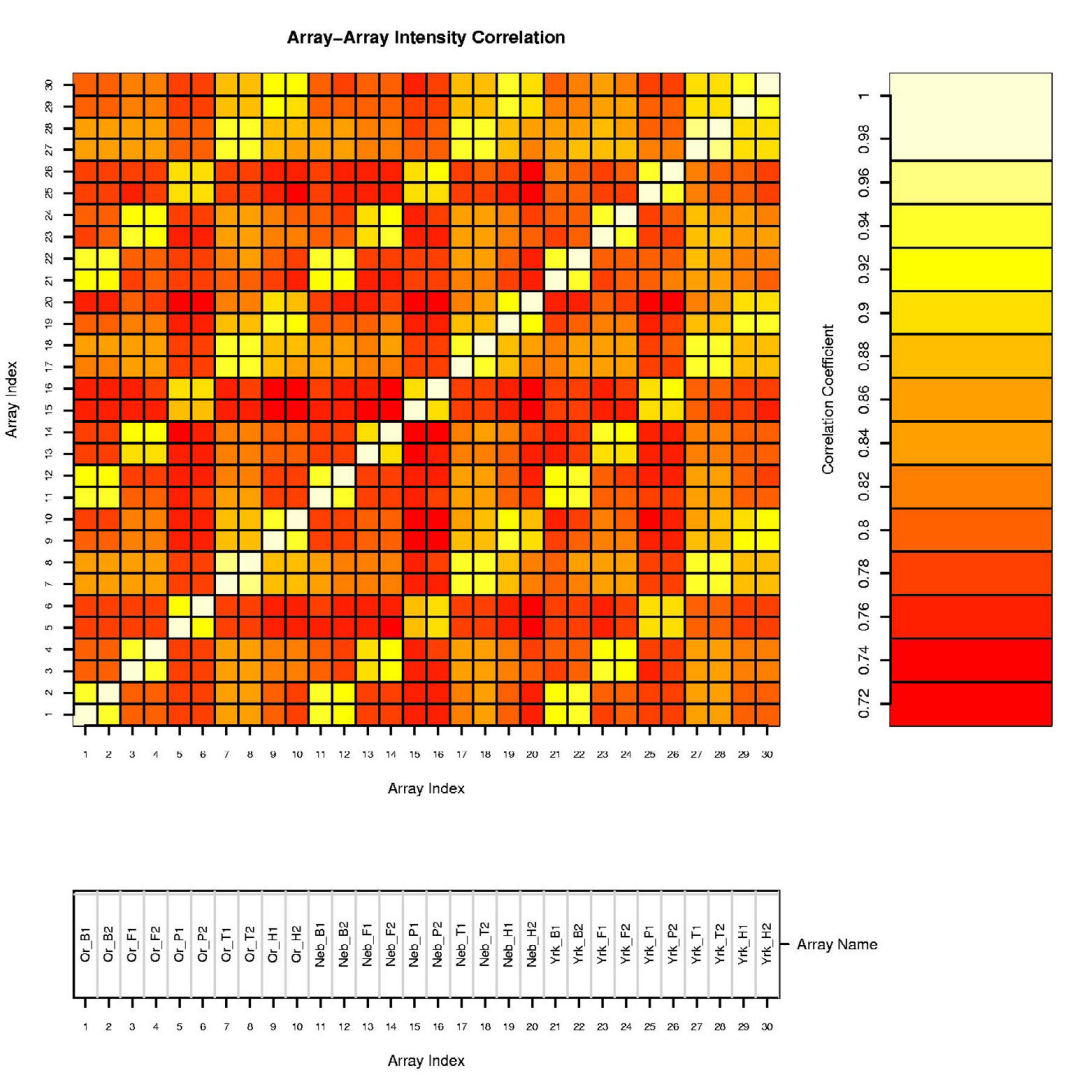
**Heat Map Plot of Array-array Correlation Coefficients. **The Spearman correlation coefficient was computed to quantify the degree of similarity between each pair of arrays. For the array name, "Or, Neb and Yrk" stands for three laboratories from the centers of Oregon, Nebraska and Yerkes, respectively while "B, F, P, T, H" represent the tissues of Cortex, Fibroblast, Pancreas, Testis and Thymus, respectively. "1 and 2" are the two replicated measurements for each RNA extract.

Correlation analysis is useful for detecting outlier arrays and can provide valuable information about phenotypic characteristics of the data (e.g., replicate, tissue). Figure 2 presents a heat map of array-array correlation coefficients for the rhesus data, where the Spearman rank correlation coefficient was computed to quantify the degree of similarity between each pair of arrays. This plot was generated using the Bioconductor library *affyQCReport *[11]. The smallest correlation coefficient was 0.72, indicating good quality of the data. It was observed that pairs of arrays had stronger correlation within tissues than correlation between tissues regardless of laboratories, showing good reproducibility of the hybridizations across three centers.

### Precision assessment

**Figure 3:**
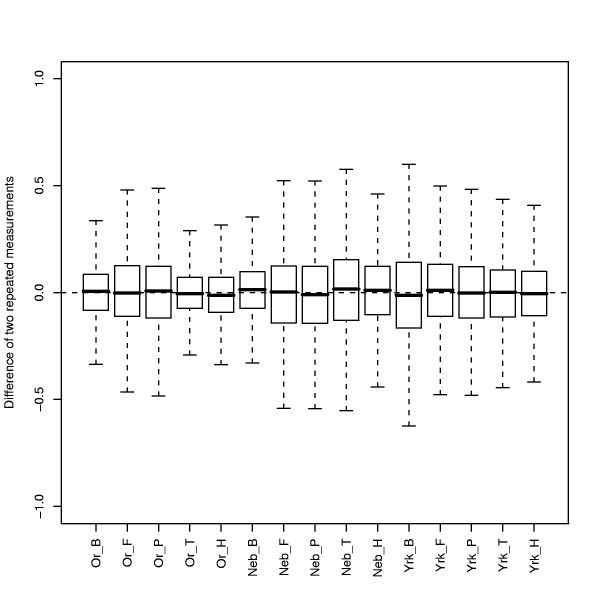
**Boxplot of the difference between sample replicates**. After RMA preprocessing, probeset signal differences between sample replicates were plotted to assesss precision. x axis: the first letters "Or, Neb and Yrk" stand for three laboratories from the centers of Oregon, Nebraska and Yerkes, respectively. The second letters "B, F, P, T, H" represent Brain (Cerebral Cortex), Fibroblast, Pancreas, Testis and Thymus, respectively.

In order to assess precision, we plotted the difference between replicate probeset signals to summarize precision for the data. This procedure was done for each tissue, from each laboratory (Figure 3). All boxplots were centered around zero and the interquartile range (IQR) values were small, indicating good precision.

### Reliability of the rhesus GeneChip at different centers

**Figure 4:**
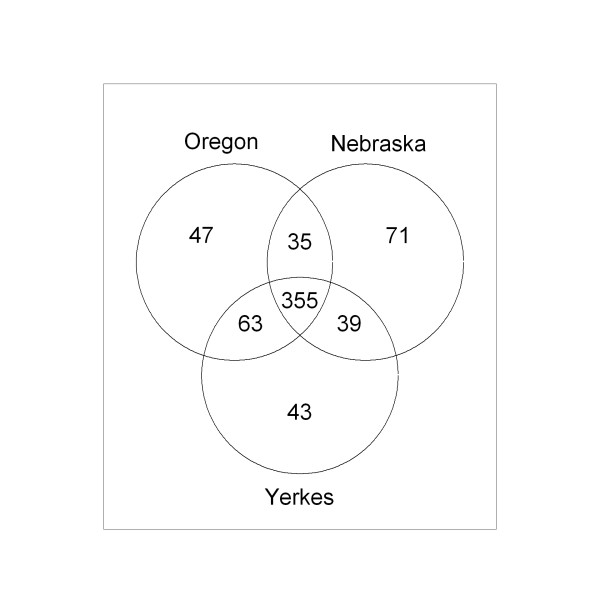
**Venn diagram of the 500 most differentially expressed genes across three laboratories. **The SAM algorithm was applied to arrays from each laboratory, preprocessed by RMA, to select the 500 most differentially expressed genes for each center. This Venn diagram shows the number of genes that were found in common among the centers.

One of the basic aims of an expression array experiment is to identify differentially expressed genes (DE genes) between samples. In this study, we conducted a "significance analysis of microarrays" (SAM) [12] to identify genes that are differentially expressed across five tissues for each laboratory. We then evaluated the agreement between laboratories in terms of the DE gene identification. Many investigators consider approximately 1% of all probesets to be differentially expressed [5,13]. We therefore examined the 500 most differentially expressed probesets out of 52,000 (figure 4). The overall agreement between laboratories is 71% (355/500). The agreement between any two laboratories is slightly higher with the values around 80% (from 0.78 to 0.84).

**Figure 5:**
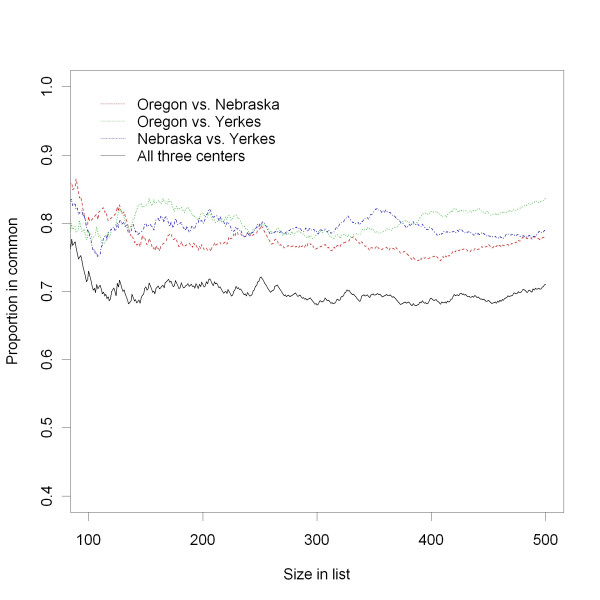
**Pairwise CAT plot. **Each of the dashed lines represents CAT curves that indicate the agreement between pairs of laboratories while the solid line indicates the agreement across alll three laboratories. These results were obtained by plotting the proportion of common genes between laboratories (Y axis) and the number of differentially expressed genes (X axis).

To further assess the reproducibility across laboratories, we drew a correspondence at the top (CAT) plot [5]. A CAT curve is created by plotting the proportion of genes in common between Centers against the number of DE genes. A CAT plot provides more information than a Venn diagram because it shows the "dynamic" agreement between two or more Centers. Figure 5 displays the CAT plot of DE genes for the three Centers. The values of agreement at the sizes 100, 200, 300, 400 and 500 are 0.73, 0.71, 0.68, 0.69 and 0.71, respectively.

### Validation of the rhesus GeneChip

**Figure 6:**
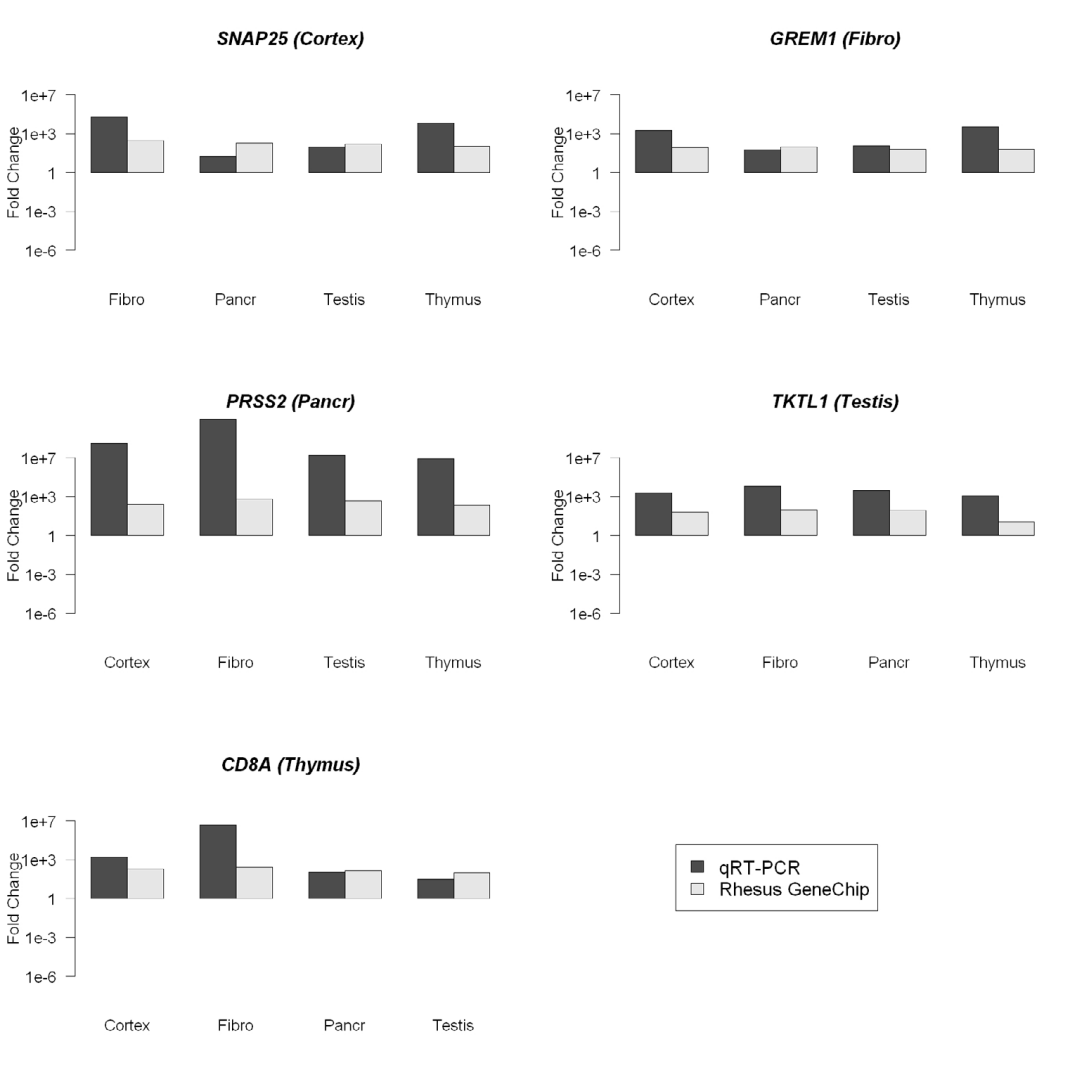
**Agreement within genes for fold changes determined by Affymetrix rhesus GeneChip and quantitative real-time PCR. **The "rhesus-array Fold Change" is defined as: Mean Signal of the target gene in its exclusively-expressed tissue/Mean Signal of the target gene in the other tissue, while the gene signal was estimated from the Affymetrix rhesus arrays. The "qRT-PCR Fold change" is defined as: 2^ΔΔCt ^ΔΔCt = (Ct of the target gene in its exclusively-expressed tissue – Ct of the control gene in its exclusively-expressed tissue) – (Ct of the target gene in the other tissue – Ct of the control gene in the other tissue), while the threshold cycle (Ct) was the average value from five replicates from the qRT-PCR assay. Please note the Ct value of the gene PRSS2 in the tissue of Fibroblast was missing due to the undetermined readings. Thus, the maximum cycle number 40 was assigned to approximate the value.

To validate the rhesus macaque GeneChip using a different measure of gene expression, five genes, each of which was highly expressed in one tissue, were chosen for quantitative real-time PCR (qRT-PCR) analysis (Figure 6). A gene's relative expression (e.g., fold change measurements between two tissues) is commonly used as the metric for the validation purpose [5,14,15].

The performance of the Affymetrix rhesus array and the qRT-PCR assay were compared in terms of each gene's relative expression (i.e., log2 fold change) between tissues (Figure 6). For each selected gene, the fold change represents the ratio of the expression of the gene in its highly-expressed tissue to its expression in each of the other tissues. All fold change measurements from the qRT-PCR assay were considerably larger than 0, indicating each of the selected genes was highly expressed in one of five tissues.

For all 5 genes, the qPCR and GeneChip results were in agreement with regard to differential expression between tissues. With respect to each individual gene, on the average, the largest fold change measurements from the Affymetrix rhesus array occurred in the gene PRSS2, which was also consistent with the results from the qRT-PCR results. In most cases, the fold change measurements from the qRT-PCR assay were larger than the corresponding ones from the Affymetrix rhesus array, which is often observed [16,17].

### Comparison of the rhesus GeneChip with the human GeneChip for studying gene expression in rhesus macaque samples

**Figure 7:**
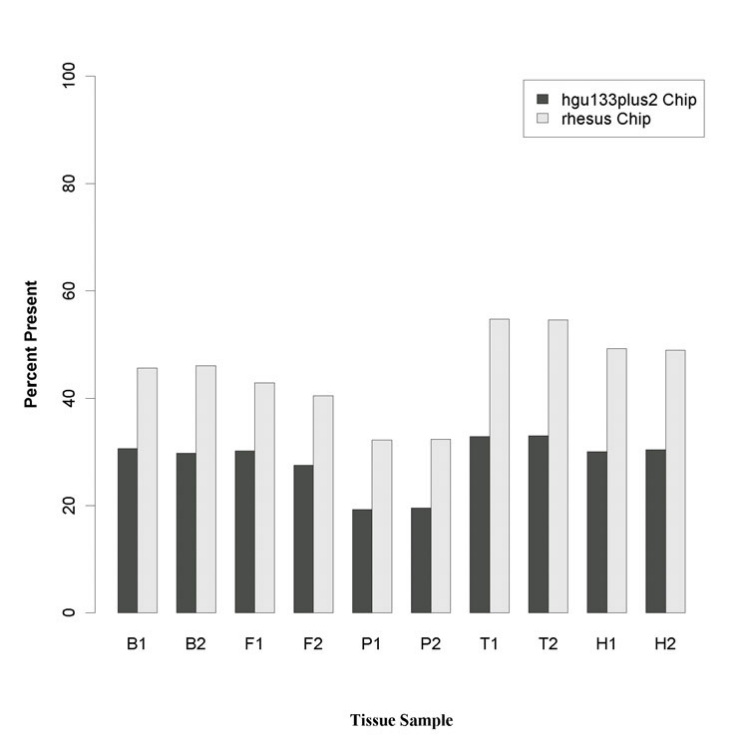
**Comparison of Percent Present calls of rhesus samples hybridized with human and rhesus macaque GeneChips. **Percent Present called after hybridization of the rhesus samples with the human Affymetrix GeneChip (hgu133plus2) (dark bars) and the rhesus macaque GeneChip (light bars). Y – axis indicates the percent of total probesets scored present using the standard Affymetrix default parameters. X – axis indicates the results obtained with each rhesus macaque sample. "B, F, P, T, H" represent Brain (Cerebral Cortex), Fibroblast, Pancreas, Testis and Thymus, respectively. Numbers represent each of two replicates for each tissue sample.

To determine how the rhesus GeneChip compares with a human GeneChip for detecting gene expression in rhesus samples, two samples (each) from the same rhesus macaque sources described above (cerebral cortex, fibroblast, pancreas, testis and thymus) were hybridized with the Affymetrix human (HGU133plus2.0) GeneChip. For every tissue and replicate, there was a much higher Percent Present call with the rhesus macaque GeneChip than with the human GeneChip (figure 7). The mean difference between the Percent Present calls with the rhesus macaque and human GeneChips was significant at the .001 level.

## Discussion

We assessed the reliability of the Affymetrix Rhesus Macaque GeneChip, between three laboratories using five tissues. The reliability of the Rhesus GeneChip was excellent; similar to the reported reliability of the human GeneChip [4]. This is important because macaques are in short supply and quite expensive. The proven reliability of the macaque GeneChip will facilitate large study designs utilizing animals from different centers.

We also assessed the validity of the rhesus GeneChip by comparing the expression patterns of a group of genes that were found to be differentially expressed in the GeneChip experiments with a qRT-PCR assay. The results indicate that the rhesus GeneChip yields accurate and valid data. This is important because the design of this GeneChip was unique. The sequences that were used to choose the probes were almost all genomic. The results from this study validate the idea that, in closely related species, probes from last exons can be successfully used. Exhaustive, expensive EST projects are not necessary to create genome wide gene chips when last exons of the target species can be identified.

The development of a reliable, valid rhesus macaque GeneChip should facilitate many important studies. Our results indicate that using the rhesus GeneChip with rhesus samples represents a significant improvement over use of the human GeneChip. It is expected that researchers in the fields of AIDS, vaccine development, transplant biology, stem cell/reproductive biology and the neurosciences will be able to use the rhesus macaque GeneChip to make important breakthroughs.

## Conclusion

The rhesus macaque GeneChip is reliable across centers and is a valid tool for measuring gene expression. It offers significant advantages in sensitivity as compared to the human GeneChip for expression analysis.

## Methods

### RNA isolation and GeneChip hybridization

The RNA samples were extracted from four tissues and one cell line of rhesus macaques: immortalized fibroblasts (Nebraska), cerebral cortex (Yerkes), pancreas (Oregon), testis (Oregon) and thymus (Oregon). Two aliquots of each RNA sample were distributed to each of three centers: Oregon National Primate Research Center, Yerkes National Primate Research Center and the University of Nebraska Medical Center. Each center labeled and hybridized samples using standard Affymetrix protocols. The Rhesus Macaque Genome GeneChip that consists of over 52,000 probe sets (Affymetrix, Santa Clara, CA) was used for all hybridizations. A total of 30 hybridizations were performed (three centers x five samples x two replicates) using Affymetrix reagents (Affymetrix). In addition, two aliquots of each sample were hybridized with the human GeneChip (HGU133plus2.0).

Total RNA was extracted from these tissues using Trizol Reagent (Invitrogen, Carlsbad, CA) as described by the manufacturer: fibroblast, pancreas, thymus, testis, and cerebral cortex. RNA was treated with DNase 1 (Invitrogen) and followed by a cleanup procedure using Qiagen RNeasy mini kit (Qiagen, Valencia, CA) according to the manufacturer's protocols. The RNA quality was assessed using an Agilent 2100 Bioanalyzer and RNA 6000 Nano LabChips (Agilent, Palo Alto, CA). All RNA samples showed a 260/280 ratio between 1.8 and 2.0 and 28S:18S ratio of 1.5 and higher. Target RNA labeling, hybridization and post-hybridization processing were performed following the Affymetrix GeneChip Expression Analysis manuals (Affymetrix). The 5 μg of RNA sample was first reverse-transcribed using T7-Oligo(dT) Promoter Primer and SuperScript II in the first-strand cDNAs synthesis reaction. Following RNase H-mediated second-stranded cDNA synthesis, the double-stranded cDNAs were purified by use of a GeneChip sample clean-up module and served as templates in the generation of biotinylated complementary RNAs (cRNAs) in the presence of T7 RNA Polymerase and a biotinylated nucleotide analog/ribonucleotide mix by in vitro transcription (IVT) reaction. The biotinylated cRNAs were cleaned up, fragmented, and hybridized to the rhesus macaque expression arrays at 45°C for 16 h with constant rotation at 60 rpm. The microarrays were washed and stained with Affymetrix fluidics stations and scanned on Affymetrix scanner 3000. The images were processed to collect raw data with GeneChip Operating Software (GCOS).

### Affymetrix GeneChip preprocessing

We exported .cel files from Affymetrix GCOS software and preprocessed them with robust multiarray analysis (RMA) [18]. Three steps were taken to process the probe-level data of the Affymetrix oligo arrays: background correction, normalization and probe-level summarization at the log2-scale. Instead of the default RMA background correction, we used MAS 5.0 (Affymetrix) to correct for background because, in our experience, probe-level intensities are more normally distributed after MAS 5.0 background correction. Quantile normalization was applied to arrays from each Center separately to control for variation in hybridizations at the different Centers. Intensity values from all 30 arrays were combined and summarized by RMA to extract probeset-level data. Data quality was assessed using the *affy *Bioconductor package [19]. The raw data (30 .CEL files) has been uploaded to th GEO repository [20] (GEO accession number is GSE7094).

### Quality assessment

Various quality assessment measures were calculated to assess the quality of the experiments, some of which were outputted from Affymetrix GCOS/MAS 5.0, such as the background levels, the range of the present percentage, the scaling factor values, the 3'/5' ratios of housekeeping genes (e.g., β-actin, GAPDH), and the signal intensities of spike-in hybridization controls. Two byproducts from RMA --- RNA degradation indices and NUSE values --- were also used to assess the quality of the experiments [21].

The scaling factor is the amount of scaling applied to the values for each array to make its intensity mean equal to a pre-specified value, which is called Target intensity value (TGT); the default value is usually 100. In practice, the trimmed (e.g., 0.02) intensity mean is commonly computed for each experiment. The assumption behind SF is that gene expression does not change significantly for the vast majority of transcripts in an experiment. Affymetrix suggests that the SF values for the arrays should be within 3-fold of each another. There are a variety of reasons for an array's SF value to deviate from the others, such as the quality and amount of starting material, issues with RNA labeling and scanning, and issues with array manufacture.

NUSE is calculated by standardizing RMA-estimated standard errors across arrays. In this way, the differences in variability between probesets can be adjusted and the boxplot of these standardized values can be used to compare chip qualities [21].

### Correlation analysis

The rank-based Spearman correlation coefficient was computed to quantify the degree of similarity between each pair of arrays. The results were visualized with a heat map plot. This plot was generated using the Bioconductor library *affyQCReport*.

### Identification of differentially expressed genes

To identify genes that are differentially expressed among five tissues, we conducted "significance analysis of microarrays" (SAM) proposed by Tusher, Tibshirani and Chu [12]. SAM is a supervised learning program for cDNA and oligo microarrays. It identifies genes with statistically significant changes in expression by assimilating a set of gene-specific t-tests, while the standard deviation is adjusted by adding a small positive constant to ensure that the variance of the calculated score is independent of gene expression. To account for the multiple comparisons and overcome the difficulties of having few replicates, SAM performs a random permutation among experiments and sets up a cut-off threshold (e.g., at 5%) to identify the significant genes. For the cut-off threshold, SAM estimates the false discovery rate (FDR) by counting the number of random-permutated genes which exceeds the cut-off threshold in the original setting.

### Quantitative real-time PCR

Real time PCR reactions were performed on an ABI 7500 PCR Thermal Cycler using RQ (Relative Quantitation) software (Applied Biosystems, Foster City, CA, USA). The reaction mix contained 1 μg of cDNA, 900 nM primers (ABI) and 250 nM probe (ABI), and 25 μl of TaqMan Universal PCR Master Mix (ABI) which contains AmpliTaq Gold DNA Polymerase, giving a final volume of 50 μl. The thermal cycler conditions were as follows: two initial steps: 2 minutes @ 50°C, 10 minutes @ 95°C, Cycling conditions: 40 cycles of 15 seconds @ 95°C and 1 minute @ 60°C. All mRNA expression values were normalized to GAPDH. Primer and probe sequences for genes being screened are as follows:

All of the above primer and probe sequences were generated and prepared by ABI custom gene expression assay service using rhesus macaque mRNA sequences.

### Percent present computation

The Affymetrix MAS 5.0 algorithm was used to generate the detection call information for each probe set, where the Affymetrix default values (0.15, 0.04 and 0.06) were used for parameters tau, alpha1 and alpha2, respectively. Percent present for each array was computed as the number of probesets called Present out of the total number of probesets.

## Authors' contributions

FD analyzed the data and wrote the paper. ERS supervised data collection at the Oregon center and contributed to the writing of the paper. YHL supervised data collection at the Yerkes center and contributed to the writing of the paper. RBN organized the project, supervised data collection at the Nebraska center and contributed to the writing of the paper. All authors read and approved the final manuscript.

## Figures and Tables

**Table 1:** Scaling factors (SF) of 30 arrays hybridized to Affymetrix GeneChips. Table 1 presents the scaling factors (SF) of all 30 arrays, where SF was calculated by setting up the Target Intensity (TGT) to be 100.

Oregon	Array	Cor1	Cor2	Fib1	Fib2	Pan1	Pan2	Tes1	Tes1	Thy1	Thy2
	SF	1.29	1.19	0.96	1.22	3.60	3.54	0.91	0.95	1.05	1.04
Nebraska	Array	Br1	Br2	Fib1	Fib2	Pan1	Pan2	Tes1	Tes1	Thy1	Thy2
	SF	0.92	1.09	1.05	1.20	4.58	3.88	0.94	0.77	1.28	1.27
Yerkes	Array	Br1	Br2	Fib1	Fib2	Pan1	Pan2	Tes1	Tes1	Thy1	Thy2
	SF	1.13	0.84	0.65	1.09	1.75	2.13	0.89	0.75	0.92	0.80

Cor = cererbral cortex, Fib = fibroblast, Pan = pancreas, Tes = testis and Thy = thymus. 1 and 2 are two technical replicates.

**Table 2:** 

Gene	
SNAP25	Forward primer
	TGCTGTGAAACAGTGTGGATGTAAA
	Reverse primer
	GCAACAAAGAATTGTACTGATAACAAACCA
	Probe
	ACTAACCACCATTTCCC
TKTL1	Forward primer
	GAATTGTAGATTGCAGTAGCCTAGGT
	Reverse primer
	GGAACAGCGGTAGGTGTCATG
	Probe
	CCACAGCACAATAACA
PRSS2	Forward primer
	GCTGAGTGTGAAGCCTCCTA
	Reverse primer
	AGGAAGCCCACACAGAACATG
	Probe
	TTGCTGGTAATCTTCC
CD8A	Forward primer
	GGCAGACCTGACTCTCTTAAGGA
	Reverse primer
	CAGGCATTGCTTGTACCATACTGTA
	Probe
	CTGCTGTCCCAAACAC
GREM1	Forward primer
	TGCAGATTTGGCTCAAGTAAAGAGA
	Reverse primer
	GCTGTGCTTTTAGGGTAGTTATGCT
	Probe
	CTCCACTGGAATAACC
